# Multiparametric Radiomics for Characterization and Outcome Prediction in Colorectal Cancer: The Central Role of Diagnostic Imaging

**DOI:** 10.3390/cancers18162578

**Published:** 2026-08-11

**Authors:** David Farkas, József Baracs, Zsombor Ritter, David Sipos

**Affiliations:** 1 Dr. József Baka Diagnostic, Radiation Oncology, Research and Teaching Center, “Moritz Kaposi” Teaching Hospital, Guba Sándor Street 40, 7400 Kaposvár, Hungary; farkas.david@sic.medicopus.hu (D.F.); david.sipos@etk.pte.hu (D.S.); 2 Faculty of Health Sciences, Doctoral School of Health Sciences, University of Pécs, 7621 Pécs, Hungary; ritter.zsombor@pte.hu; 3 Department of Surgery, Medical Center, University of Pécs, 7624 Pécs, Hungary; 4 Department of Medical Imaging, Medical School, University of Pécs, 7624 Pécs, Hungary; 5 Department of Medical Imaging, Faculty of Health Sciences, University of Pécs, 7621 Pécs, Hungary

**Keywords:** colorectal cancer, radiomics, multiparametric imaging, PET/CT, MRI, CT, machine learning

## Abstract

Background/Objectives: Colorectal carcinoma (CRC) remains a leading cause of cancer-related morbidity and mortality worldwide, with increasing incidence in younger populations. Despite advances in imaging, conventional approaches remain limited by subjective interpretation and insufficient characterization of tumor heterogeneity. Radiomics, particularly in a multiparametric framework integrating computed tomography (CT), magnetic resonance imaging (MRI), and positron emission tomography (PET), has emerged as a promising tool to enhance diagnostic and prognostic performance. This review aims to critically evaluate methodological strategies for optimizing multiparametric radiomics in CRC. Methods: A narrative review was conducted based on a literature search of PubMed/MEDLINE, Scopus, and Web of Science up to March 2026. Studies focusing on CT-, MRI-, and PET-based radiomics in CRC were included. Key methodological aspects analyzed included imaging acquisition and standardization, tumor segmentation techniques, radiomic feature extraction, feature selection methods (e.g., LASSO and PCA), and model validation approaches. Results: Multiparametric radiomics models integrating CT, MRI, and PET consistently demonstrated superior diagnostic accuracy compared to single-modality approaches, particularly in T-staging and lymph node involvement prediction. PET-derived metabolic features further enhanced characterization of tumor biology and improved prognostic stratification, including prediction of progression-free survival (PFS) and overall survival (OS). However, methodological heterogeneity, small sample sizes, and variability in imaging protocols and segmentation practices remain significant limitations affecting reproducibility and generalizability. Conclusions: Multiparametric radiomics represents a powerful advancement in precision oncology for CRC, enabling improved tumor characterization, risk stratification, and personalized treatment planning. Standardization, multicentric validation, and integration with artificial intelligence are essential for successful clinical translation.

## Simple Summary

Colorectal cancer is one of the most common and deadly cancers worldwide, and its successful treatment depends heavily on early detection and accurate assessment of how far the disease has progressed. Medical imaging techniques such as computed tomography, magnetic resonance imaging, and positron emission tomography are routinely used, but their interpretation can vary between specialists and may not fully capture the complexity of tumors. This review explores a modern approach called radiomics, which extracts detailed quantitative information from medical images to better describe tumor characteristics. By combining data from multiple imaging methods and clinical information, the authors aim to identify strategies that improve diagnosis, predict patient outcomes more accurately, and support personalized treatment decisions. The findings may help guide future research toward more reliable, standardized, and clinically useful imaging-based tools.

## 1. Introduction

Colorectal carcinoma (CRC) remains a major global health burden, ranking among the most frequently diagnosed malignancies and leading causes of cancer-related mortality worldwide [1]. Its incidence demonstrates marked geographic variability, with higher rates observed in developed regions; however, a concerning increase has also been reported among younger populations across multiple countries. Despite advances in screening programs and therapeutic strategies, CRC continues to pose substantial clinical challenges because of its frequently asymptomatic early stages, heterogeneous biological behavior, and variable response to treatment [2]. Consequently, improving early detection, accurate staging, and individualized prognostic assessment remains a major priority in contemporary oncologic research and clinical practice [1,2,3].

Diagnostic imaging plays a pivotal role in the comprehensive management of colorectal cancer, encompassing detection, staging, treatment planning, and response assessment. Computed tomography (CT) is widely used as a first-line imaging modality because it provides rapid image acquisition, high spatial resolution, and reliable assessment of locoregional disease and distant metastases, particularly in the liver and lungs. Magnetic resonance imaging (MRI), especially for rectal cancer, offers superior soft-tissue contrast and is indispensable for the detailed evaluation of tumor infiltration, circumferential resection margin involvement, and nodal status [4,5]. In addition, positron emission tomography combined with computed tomography (PET/CT) provides complementary functional information by assessing tumor metabolic activity, thereby improving the detection of occult metastases, evaluation of treatment response, and identification of recurrent disease [4,5,6,7].

Despite these advances, current imaging approaches remain subject to several inherent limitations. Visual image interpretation is still largely subjective and dependent on the expertise of the interpreting radiologist, resulting in interobserver variability and potential diagnostic inconsistencies [1,2,3]. Furthermore, conventional imaging techniques may fail to adequately capture the underlying biological heterogeneity of tumors, which is increasingly recognized as a key determinant of tumor aggressiveness, treatment resistance, and clinical outcomes. This limited characterization of intratumoral complexity restricts the full exploitation of imaging data for precision medicine applications [4,5,6].

Radiomics has emerged as a transformative approach that seeks to overcome these limitations by extracting a large number of quantitative features from routinely acquired medical images, including CT, MRI, and PET/CT datasets, thereby transforming medical images into high-dimensional, mineable data [4,5,6,7,8]. These features characterize tumor morphology, texture, signal intensity, and spatial relationships, providing a non-invasive means of assessing tumor phenotype and heterogeneity beyond the limits of visual interpretation. By integrating advanced computational techniques with machine learning algorithms, radiomics has considerable potential to improve diagnostic accuracy, risk stratification, and prediction of treatment response [7,8].

The present review aims to critically evaluate the methodological approaches required to maximize the clinical utility of multiparametric radiomics in colorectal cancer. Particular emphasis is placed on best practices for image acquisition standardization, tumor segmentation, feature extraction, data integration, and model validation. Special attention is given to the integration of PET-derived functional biomarkers with CT- and MRI-derived radiomic features, thereby enabling a comprehensive multiparametric imaging framework. By synthesizing the current evidence and highlighting key methodological considerations, this review outlines strategies to improve the robustness, reproducibility, and clinical translation of radiomics while maximizing the diagnostic and prognostic value of advanced multimodal imaging in colorectal cancer.

## 2. Materials and Methods

This narrative review was designed to provide a comprehensive and critical synthesis of current methodological approaches aimed at optimizing the diagnostic and prognostic performance of multiparametric radiomics in colorectal cancer.

A systematic literature search was conducted across the major electronic databases PubMed/MEDLINE, Scopus, and Web of Science to identify relevant studies published up to March 2026. The search strategy combined controlled vocabulary and free-text keywords related to colorectal cancer (“colorectal carcinoma,” “rectal cancer,” and “colon cancer”), imaging modalities (“computed tomography,” “magnetic resonance imaging,” and “PET/CT”), and radiomics (“radiomics,” “texture analysis,” “quantitative imaging,” and “machine learning”). Boolean operators (“AND” and “OR”) were used to refine the search strategy and maximize sensitivity. In addition, the reference lists of eligible articles were manually screened to identify further relevant publications.

Eligible studies included original research articles, systematic reviews, and methodological papers investigating radiomics applications in colorectal cancer, particularly those addressing CT-, MRI-, and PET/CT-based feature extraction, multiparametric data integration, and diagnostic or prognostic model development. Studies were included if they were published in English and provided sufficient methodological detail to ensure reproducibility. Conference abstracts, case reports, editorials, and studies lacking reproducible methodological descriptions were excluded.

The database search initially identified 1247 records. After the removal of duplicates (n = 312), 935 records underwent title and abstract screening. Subsequently, 146 articles were selected for full-text review. Following full-text assessment, 52 studies met the predefined eligibility criteria and were included in the final qualitative synthesis.

Study selection was independently performed by two reviewers through title and abstract screening, followed by full-text evaluation of potentially eligible articles. Disagreements regarding study eligibility were resolved through discussion and consensus. When consensus could not be reached, a third reviewer was consulted to make the final decision.

Given the narrative nature of this review, no formal meta-analysis was performed. Instead, emphasis was placed on methodological quality, reproducibility, and clinical relevance. Data extracted from the included studies focused on imaging acquisition protocols, tumor segmentation techniques, radiomic feature extraction methods, feature selection strategies, predictive model development, validation approaches, and clinical applicability.

The evidence was synthesized qualitatively to identify methodological trends, common limitations, and best-practice recommendations for developing robust, reproducible, and clinically translatable multiparametric radiomics models in colorectal cancer.

## 3. Imaging Acquisition and Standardization

High-quality, standardized imaging constitutes the cornerstone of robust and reproducible radiomics analyses in colorectal cancer [9]. Variability in acquisition protocols, scanner settings, and image reconstruction techniques can substantially influence the extracted radiomic features, thereby limiting comparability across studies and reducing model generalizability. Consequently, the implementation of harmonized imaging protocols is essential to ensure data consistency, reproducibility, and reliability [9,10,11].

In CT, contrast-enhanced imaging is fundamental for optimal lesion characterization. Standardized multiphasic acquisition protocols, particularly those incorporating arterial and portal venous phases, are recommended [12,13]. The arterial phase facilitates the assessment of tumor vascularity and hyperenhancing lesions, whereas the portal venous phase provides superior delineation of primary tumors and improved detection of liver metastases. Consistency in contrast administration, including dose, injection rate, and timing, as well as reconstruction parameters such as slice thickness and reconstruction kernel, is critical for minimizing variability in radiomic feature extraction. In hybrid PET/CT systems, CT acquisition must also be carefully standardized because it directly influences attenuation correction and the quantitative accuracy of PET imaging [12,13,14].

MRI, particularly for rectal cancer, requires a multiparametric approach to capture the complex biological characteristics of the tumor. Standard imaging protocols typically include high-resolution T2-weighted sequences for anatomical delineation, T1-weighted imaging for structural assessment, and diffusion-weighted imaging (DWI) with corresponding apparent diffusion coefficient (ADC) maps to evaluate tissue cellularity and microstructural properties [15,16]. The integration of these complementary sequences enables comprehensive characterization of both tumor morphology and biological behavior. Strict adherence to acquisition parameters—including magnetic field strength, b-values for DWI, slice orientation, and spatial resolution—is essential to ensure reproducibility and facilitate reliable radiomic analyses [15,16,17]. Emerging hybrid PET/MRI systems further extend this paradigm by enabling the simultaneous acquisition of metabolic and high-resolution soft-tissue information, thereby enriching multiparametric radiomic feature sets [18].

PET/CT provides complementary functional information by quantifying tumor metabolic activity, most commonly using ^18^F-fluorodeoxyglucose (^18^F-FDG), thereby supporting disease staging, treatment response assessment, and the detection of recurrent disease. Standardization of PET imaging is equally important and includes harmonization of uptake time, administered tracer dose, image reconstruction algorithms, and quantitative metrics such as standardized uptake values (SUVs). PET-derived radiomic features provide valuable information on tumor metabolism and biological aggressiveness, complementing anatomical imaging modalities and enhancing the performance of multiparametric radiomics models [19,20].

**Figure 1:**
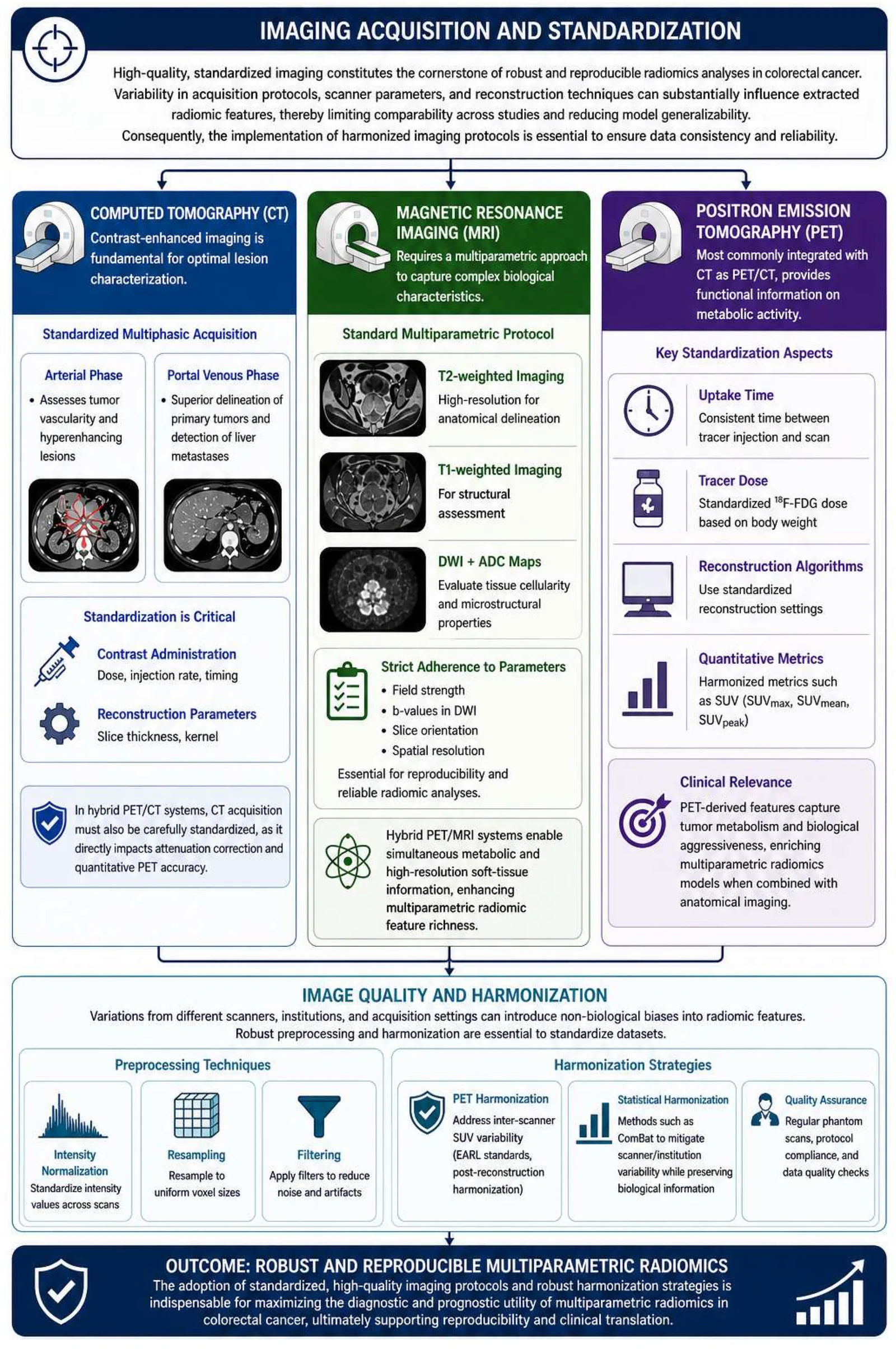
Overview of standardized imaging acquisition protocols, preprocessing techniques, and harmonization strategies across CT, MRI, and PET/CT for multiparametric radiomics in colorectal cancer.

Beyond image acquisition, image quality assurance and harmonization represent critical methodological considerations. Variations introduced by different scanners, institutions, acquisition protocols, and reconstruction settings can introduce non-biological bias into radiomic features, thereby reducing their reproducibility. To minimize these effects, preprocessing techniques such as intensity normalization, resampling to uniform voxel sizes, and image filtering are routinely applied to standardize imaging datasets. In PET imaging, harmonization is particularly important because of inter-scanner variability in SUV measurements. Consequently, standardized approaches such as the European Association of Nuclear Medicine Research Ltd. (EARL) accreditation standards and post-reconstruction harmonization techniques are increasingly implemented. Furthermore, advanced statistical harmonization methods, including ComBat, have gained widespread acceptance for reducing inter-scanner variability while preserving biologically relevant information [21,22] (Figure 1).

## 4. Tumor Segmentation

Accurate tumor segmentation represents a critical step in the radiomics workflow, as it defines the region from which quantitative imaging features are extracted. Consequently, the reliability, reproducibility, and biological validity of radiomic analyses are highly dependent on the accuracy and consistency of segmentation methodologies across imaging modalities, including CT, MRI, and PET [23,24].

Segmentation approaches reported in the literature can be broadly classified as manual, semi-automated, or artificial intelligence (AI)-based methods [23,24,25,26]. Manual segmentation, typically performed by experienced radiologists, remains the most widely used technique and is often regarded as the reference standard because it incorporates expert anatomical and pathological knowledge [25,26]. However, it is inherently time-consuming and susceptible to operator-dependent variability. Semi-automated approaches reduce the workload by combining algorithm-based boundary detection with manual refinement, thereby improving efficiency while maintaining expert oversight. More recently, fully automated AI-based segmentation methods, particularly those based on deep learning architectures, have gained increasing attention because of their scalability, reproducibility, and potential to standardize the segmentation process across large imaging datasets [26]. In PET imaging, segmentation may additionally rely on standardized uptake value (SUV)-based thresholding or gradient-based techniques to delineate metabolically active tumor regions, which may differ from anatomically defined tumor volumes identified on CT or MRI [18,19,20,21,22,23,24,25,26].

Despite these advances, interobserver variability remains a significant methodological challenge. Differences in contour delineation among observers can lead to substantial variability in extracted radiomic features, ultimately affecting model robustness, reproducibility, and clinical applicability [25,26,27]. This variability is particularly pronounced in tumors with poorly defined margins, heterogeneous imaging characteristics, or post-treatment changes and may be further amplified when multimodal imaging datasets are integrated. In PET-based segmentation, additional variability may arise from differences in threshold selection, image reconstruction parameters, and image noise, all of which influence the delineation of metabolic tumor volume (MTV) [25,26,27,28]. To address these challenges, several strategies have been proposed, including standardized segmentation protocols, observer training and calibration, and the use of consensus or multi-reader annotations. Furthermore, quantitative assessment of segmentation reproducibility using metrics such as the Dice similarity coefficient and intraclass correlation coefficients (ICCs) is increasingly recommended to ensure methodological rigor [28,29].

In this context, the integration of AI-based segmentation methods with rigorous validation and quality assurance procedures holds considerable promise for reducing interobserver variability and improving the reproducibility of radiomics studies. Multimodal segmentation frameworks that simultaneously integrate CT, MRI, and PET information may further enhance segmentation accuracy by leveraging complementary anatomical and functional tumor characteristics. Therefore, establishing standardized, reproducible, and well-validated segmentation workflows is essential for the successful clinical translation of multiparametric radiomics in colorectal cancer [19,20,21,25,26,27,28].

**Figure 2:**
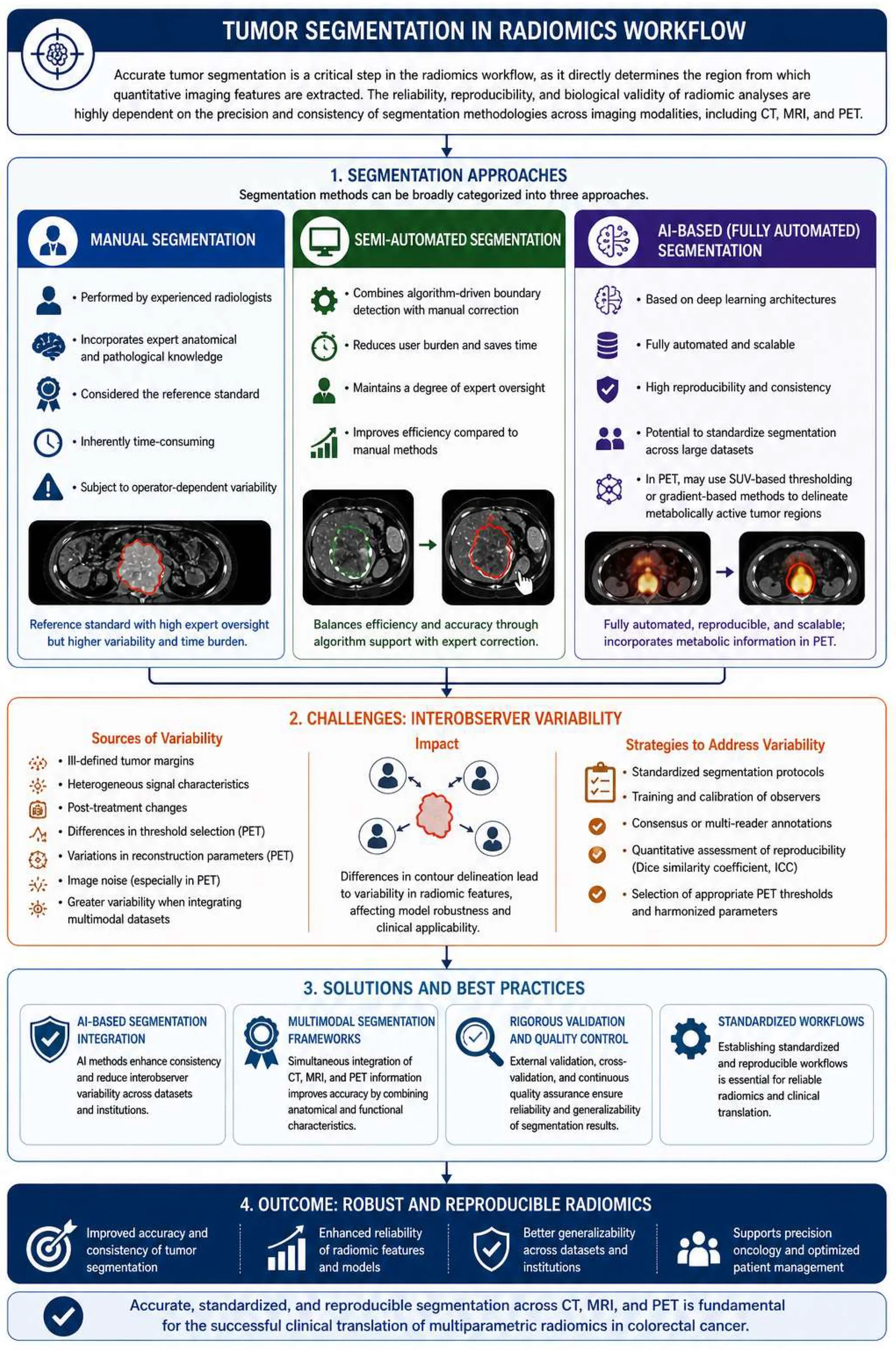
Comprehensive overview of manual, semi-automated, and AI-based tumor segmentation methods, interobserver variability, and quality assurance strategies for reliable radiomic feature extraction in colorectal cancer.

The principal segmentation approaches, sources of variability, and harmonization strategies used in multiparametric radiomics are summarized in Figure 2.

## 5. Radiomic Feature Extraction

Radiomic feature extraction is a fundamental step in transforming medical images into quantitative data capable of capturing tumor phenotype and underlying biological heterogeneity. These features are typically derived from segmented tumor volumes across imaging modalities, including CT, MRI, and PET, and can be broadly classified into first-order statistics, texture features, shape descriptors, and higher-order features generated through mathematical transformations [7,8,21,22,29].

First-order statistics describe the distribution of voxel intensities within the segmented tumor volume without considering spatial relationships. These features include metrics such as mean, median, standard deviation, skewness, kurtosis, entropy, and energy. They provide a global summary of image intensity and are often associated with tumor density, cellularity, and the presence of necrotic components. In PET imaging, first-order features are commonly derived from standardized uptake values (SUVs), including SUVmean, SUVmax, and SUVpeak, reflecting tumor metabolic activity and glycolytic burden. Although relatively simple, first-order features provide valuable baseline information and are generally less sensitive to segmentation variability than more complex radiomic descriptors. However, several studies have reported limited prognostic performance when these features are used in isolation, suggesting that they may not adequately capture the spatial complexity of tumor heterogeneity. Furthermore, differences in image acquisition protocols, reconstruction algorithms, and intensity normalization strategies have contributed to inconsistent findings across studies, thereby limiting their generalizability [30,31].

Texture features quantify spatial relationships among voxel intensities, thereby capturing intratumoral heterogeneity beyond the limits of visual image interpretation. Among the most widely used texture analysis methods are the Gray-Level Co-occurrence Matrix (GLCM) and the Gray-Level Run Length Matrix (GLRLM). GLCM-based features evaluate the frequency of co-occurring intensity values within a defined spatial relationship, generating metrics such as contrast, correlation, homogeneity, and dissimilarity. In contrast, GLRLM features characterize consecutive runs of voxels with identical intensity values and generate parameters such as short-run emphasis, long-run emphasis, and gray-level non-uniformity [32,33]. These texture features are particularly valuable for assessing tumor aggressiveness and microstructural complexity [32]. In PET-based radiomics, texture features derived from metabolic activity maps further enhance the characterization of functional heterogeneity that may not be apparent on anatomical imaging alone. Despite their widespread use, texture features often exhibit limited reproducibility because they are influenced by image resolution, preprocessing techniques, segmentation methods, and scanner characteristics. Consequently, different studies frequently identify different texture features as significant predictors, making direct comparison and external validation challenging [30,31,32].

Shape features describe the three-dimensional geometric characteristics of the tumor independently of image intensity values. These include volume, surface area, compactness, sphericity, elongation, and irregularity. Shape descriptors are particularly useful for evaluating tumor growth patterns and local invasiveness and have demonstrated prognostic relevance across several oncologic settings [14,15,16,17,18,19,20,21,22]. In PET imaging, shape-related parameters such as MTV and TLG combine volumetric and metabolic information, providing additional biologically meaningful biomarkers [25,26,27,28]. Importantly, shape features are generally less affected by variations in image intensity than first-order or texture features but remain highly dependent on accurate tumor segmentation. Although several studies have demonstrated significant associations between tumor morphology and clinical outcomes, others have reported limited incremental predictive value after adjustment for established clinical and pathological variables [25,26,27,28,29,30,31,32].

Higher-order features are generated through mathematical transformations, such as wavelet decomposition, which enables multiscale analysis of imaging data. Wavelet transforms decompose the original image into multiple frequency components, allowing for the extraction of features that emphasize both fine and coarse textural patterns across different spatial scales [33,34,35]. This approach enhances the detection of subtle tumor heterogeneity and has been shown to improve the predictive performance of radiomics models when combined with conventional radiomic features. When applied to PET data, wavelet-based transformations can reveal multiscale metabolic heterogeneity, further enriching multiparametric radiomic signatures. However, higher-order features are particularly susceptible to overfitting because they substantially increase feature dimensionality, whereas most colorectal cancer radiomics studies remain limited by relatively small sample sizes [28,29,30,31,32,33,34,35].

## 6. Multiparametric Modelling

The development of multiparametric models represents a major advancement in radiomics by integrating complementary information from multiple imaging modalities and clinical data sources to improve diagnostic accuracy and prognostic stratification in colorectal cancer [14,30]. Given the inherent complexity and heterogeneity of tumor biology, reliance on a single imaging modality provides only a partial representation of disease characteristics. Consequently, combining features derived from CT, MRI, PET and relevant clinical variables enables a more comprehensive and biologically meaningful assessment of the disease [5,15,28,31].

Within this framework, CT provides robust information on tumor morphology, enhancement patterns, and metastatic spread, particularly to the liver and lungs. MRI, especially for rectal cancer, offers superior soft-tissue contrast together with functional information through sequences such as DWI and ADC mapping, which reflect tissue cellularity and microstructural organization [5,15,24]. PET imaging, most commonly performed as PET/CT, provides complementary metabolic information by quantifying tumor glucose uptake and biological activity, thereby capturing aspects of tumor aggressiveness and viability that cannot be assessed using anatomical imaging alone. The incorporation of clinical variables—including patient demographics, laboratory findings, histopathological characteristics, and TNM stage—further strengthens these models by integrating well-established prognostic factors [14,18,36]. The synergistic integration of these heterogeneous data sources has consistently been shown to improve predictive performance compared with unimodal approaches [21,22].

The high dimensionality of radiomic data, however, necessitates robust feature selection techniques to reduce redundancy, minimize overfitting, and improve model interpretability. Among the most widely used approaches is the Least Absolute Shrinkage and Selection Operator (LASSO), a regularization method that simultaneously performs variable selection and coefficient shrinkage. By penalizing the absolute magnitude of regression coefficients, LASSO retains only the most informative features, thereby enhancing model stability and generalizability [37,38]. This approach is particularly valuable in multiparametric models that combine PET-derived metabolic features with CT- and MRI-derived radiomic features, further increasing feature dimensionality [15,24].

Another frequently applied technique is Principal Component Analysis (PCA), a dimensionality reduction method that transforms the original feature space into a smaller set of orthogonal components that capture the maximum variance within the dataset. PCA reduces feature redundancy and multicollinearity while preserving the most informative data, thereby facilitating more efficient model development and improving computational performance. In multimodal imaging datasets that integrate CT, MRI, and PET features, PCA is particularly useful for combining heterogeneous data into a unified analytical framework [39,40].

The integration of multimodal imaging features—including CT, MRI, and PET—with clinical variables, together with rigorous feature selection strategies such as LASSO and PCA, is fundamental to the development of robust and generalizable multiparametric radiomics models. Nevertheless, the current evidence should be interpreted with caution because many published studies are retrospective, single-center investigations characterized by relatively small sample sizes and heterogeneous methodologies. Although multiparametric approaches consistently demonstrate theoretical and statistical advantages over unimodal models, their true clinical benefit remains uncertain owing to the lack of standardized imaging workflows, prospective multicenter validation, and direct head-to-head comparisons between competing modeling strategies. Addressing these limitations will be essential to facilitate the clinical translation of multiparametric radiomics and to realize its full potential for improving diagnosis, individualized risk stratification, and treatment decision-making in colorectal cancer [24,37,38,39,40].

## 7. Diagnostic Performance

**Figure 3:**
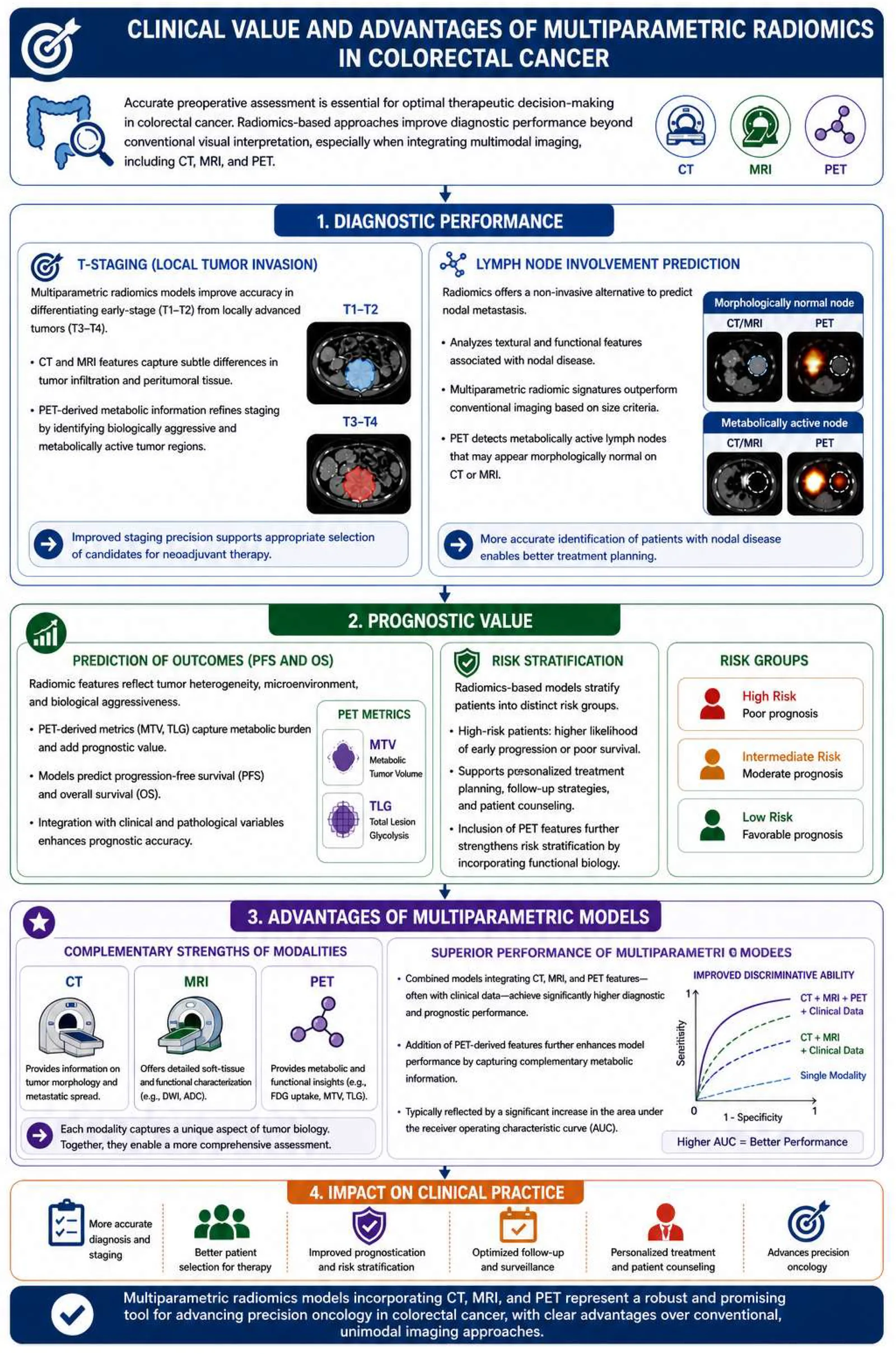
Conceptual overview of the diagnostic, prognostic, and clinical applications of multiparametric radiomics integrating CT, MRI, and PET/CT, highlighting its role in tumor staging, lymph node assessment, outcome prediction, risk stratification, and personalized management of colorectal cancer.

Accurate preoperative assessment is essential for optimal therapeutic decision-making in colorectal cancer, particularly when determining the extent of local tumor invasion and lymph node involvement. Radiomics-based approaches have demonstrated considerable potential to improve diagnostic performance beyond conventional visual image interpretation, especially through the integration of multimodal imaging data from CT, MRI, and PET [15,17,19,20]. The principal diagnostic, prognostic, and clinical applications of multiparametric radiomics in colorectal cancer are summarized in Figure 3. Reported performance metrics, however, vary substantially across studies because of differences in imaging protocols, segmentation techniques, feature extraction workflows, and patient populations. Consequently, direct comparison of published models remains challenging, and their generalizability has yet to be fully established.

With respect to T staging, multiparametric radiomics models have demonstrated improved accuracy in differentiating early-stage (T1–T2) from locally advanced tumors (T3–T4). By integrating quantitative features derived from CT and MRI, these models can detect subtle differences in tumor infiltration and peritumoral tissue characteristics that may not be readily appreciated using conventional imaging assessment [18,28]. The addition of PET-derived metabolic information may further improve staging accuracy by identifying biologically aggressive tumor regions and metabolically active tumor margins. Improved staging precision is particularly important for selecting patients who are most likely to benefit from neoadjuvant therapy. Nevertheless, the available evidence remains inconsistent. While some studies have reported significant improvements in the detection of locally advanced disease using multiparametric models, others have demonstrated only modest benefits over expert radiological assessment. Moreover, most published studies are retrospective and involve relatively small patient cohorts, limiting the robustness and generalizability of their findings. The incremental value of PET-derived features in addition to high-quality MRI also remains uncertain [28,30].

Accurate prediction of lymph node involvement remains a major clinical challenge because conventional size-based imaging criteria are often insufficient and unreliable [41,42]. Radiomics offers a non-invasive alternative by analyzing quantitative textural and functional imaging features associated with nodal metastasis. Several studies have demonstrated that radiomic signatures, particularly those derived from multiparametric imaging, achieve superior predictive performance compared with conventional imaging, enabling more accurate identification of patients with nodal disease [21,22,29,30]. In this setting, PET imaging may provide additional diagnostic value by identifying metabolically active lymph nodes that appear morphologically normal on CT or MRI. Despite these promising findings, the predictive performance of radiomics models varies considerably across studies, and only a limited number have undergone external validation. Model performance may be influenced by differences in segmentation methodology, image quality, scanner characteristics, and feature extraction pipelines, while several investigations have reported reduced accuracy when models were applied to independent patient cohorts. Consequently, although radiomics has the potential to complement conventional imaging assessment, further prospective multicenter studies with standardized methodologies are required before routine clinical implementation can be recommended [18,28].

### 7.1. Prognostic Value

Beyond diagnosis, radiomics has demonstrated considerable potential for prognostic modeling, particularly in predicting progression-free survival (PFS) and overall survival (OS) [43]. Quantitative imaging features that reflect tumor heterogeneity, the tumor microenvironment, and biological aggressiveness have consistently been associated with clinical outcomes across multiple studies. In particular, PET-derived biomarkers, such as MTV and TLG, have shown additional prognostic value by quantifying the metabolic burden of the tumor [43,44].

Radiomics-based models can stratify patients into distinct risk categories, thereby identifying individuals with an increased likelihood of early disease progression or poor survival outcomes. This capability has important implications for personalized treatment planning, individualized follow-up strategies, and patient counseling. Furthermore, integrating imaging-derived radiomic features with established clinical and pathological variables improves prognostic performance and supports a more personalized approach to patient management. The incorporation of PET-derived features into multiparametric models may further enhance risk stratification by capturing complementary aspects of functional tumor biology [18,23,43,44].

### 7.2. Advantages of Multiparametric Models

A recurring observation across the literature is that multiparametric radiomics models frequently outperform models based on a single imaging modality; however, the magnitude of this improvement varies considerably between studies. While CT provides valuable information on tumor morphology and metastatic spread, and MRI offers detailed soft-tissue characterization together with functional assessment, each modality captures only specific aspects of tumor biology [5,6,18]. PET complements these imaging techniques by providing metabolic and functional information that may further improve the characterization of tumor heterogeneity and biological aggressiveness [30].

Several studies have demonstrated that models integrating CT- and MRI-derived radiomic features, often in combination with clinical variables, achieve improved diagnostic and prognostic performance compared with unimodal approaches. Furthermore, the incorporation of PET-derived features has been associated with additional improvements in predictive performance in selected patient cohorts. However, reported performance metrics vary substantially across studies, with differences in the area under the receiver operating characteristic curve (AUC), sensitivity, and specificity likely reflecting variations in patient populations, imaging protocols, segmentation methods, feature selection strategies, and validation procedures. Consequently, direct comparison between published studies remains challenging [5,6,18]. Although several investigations have reported statistically significant improvements in AUC following the integration of multimodal imaging features, the magnitude and clinical relevance of these gains remain inconsistent across the literature [45,46].

The reported improvements in predictive performance highlight the potential value of multimodal data integration by enabling a more comprehensive characterization of tumor phenotype and biological behavior. Consequently, multiparametric radiomics models integrating CT, MRI, PET, and relevant clinical variables represent a promising strategy for advancing precision oncology in colorectal cancer. Nevertheless, the available evidence should be interpreted with caution because methodological heterogeneity, limited sample sizes, and the lack of standardized radiomics workflows continue to hinder robust comparison, external validation, and clinical translation. Future prospective multicenter studies employing standardized methodologies and rigorous external validation are essential to determine the true incremental value of multiparametric radiomics for colorectal cancer management [5,6,45,46]. The main characteristics, methodological approaches, and key findings of the original studies included in this review are summarized in the Supplementary Material.

Figure 3 illustrates the principal diagnostic, prognostic, and clinical applications of multiparametric radiomics in colorectal cancer.

## 8. Discussion

Diagnostic imaging occupies a central role in the management of colorectal cancer, extending far beyond its traditional function of anatomical visualization [4]. Advances in medical imaging technologies and computational image analysis have transformed imaging into a powerful source of quantitative biomarkers capable of characterizing tumor biology in vivo. Contemporary imaging modalities not only depict anatomical structures but also provide insights into tumor phenotype, intratumoral heterogeneity, the tumor microenvironment, angiogenesis, cellular density, and metabolic activity. In this context, radiomics represents a paradigm shift by transforming routinely acquired imaging data—including CT, MRI, and PET-derived metabolic imaging—into high-dimensional, mineable information that can support both diagnostic and prognostic decision-making [5,6,7,31].

The fundamental principle of radiomics is that medical images contain a wealth of quantitative information beyond what can be appreciated through visual interpretation alone. Using advanced computational algorithms, hundreds to thousands of quantitative features can be extracted from segmented tumor regions, including shape descriptors, intensity distributions, texture characteristics, and higher-order statistical features. These imaging biomarkers may reflect underlying biological processes and molecular characteristics that are not accessible through conventional radiological assessment. Consequently, radiomics has emerged as a promising bridge between diagnostic imaging and precision oncology, offering opportunities for non-invasive tumor characterization, risk stratification, prediction of treatment response, and personalized therapeutic planning [5,6,7,31].

Despite these advances, the clinical implementation of radiomics remains an evolving process. Although numerous studies have reported encouraging results, the successful translation of radiomics into routine clinical practice requires robust standardization, reproducibility, and external validation. Key challenges include harmonizing imaging protocols across CT, MRI, and PET, ensuring consistent tumor segmentation, and establishing transparent and interpretable model development strategies [29,30,31]. Additional sources of variability include differences in scanner hardware, acquisition protocols, reconstruction algorithms, and image preprocessing procedures, all of which can substantially influence radiomic feature values. Furthermore, the lack of universally accepted reporting standards and the limited availability of large external validation datasets have hindered comparisons across studies and institutions. Importantly, current evidence suggests that methodological quality may have a greater influence on model performance than the number of imaging modalities incorporated. Studies employing standardized imaging protocols, rigorous feature selection, and external validation generally report more robust and reproducible findings than those relying solely on increasingly complex multimodal models [23,28,30].

Nevertheless, when appropriately implemented, radiomics has the potential to complement conventional radiological assessment by providing objective quantitative biomarkers that reduce subjectivity and increase diagnostic confidence. The incorporation of PET imaging further strengthens this framework by adding complementary functional and metabolic information to anatomical and structural imaging data [6,14,19,44]. PET-derived parameters can characterize biological processes such as glucose metabolism, cellular proliferation, and tumor aggressiveness, thereby providing information that may not be evident on purely anatomical imaging. The integration of metabolic, functional, and morphological information within a single analytical framework represents one of the principal advantages of multiparametric radiomics and closely aligns with the goals of precision medicine. However, the incremental contribution of PET remains inconsistent. While several studies have reported improved diagnostic or prognostic performance following the incorporation of PET-derived features, others have demonstrated only modest gains compared with high-quality CT- and MRI-based models, suggesting that the clinical value of PET depends on the specific clinical indication and patient population [30,31,44].

The findings synthesized in this review are consistent with the growing body of evidence supporting the added value of multiparametric radiomics. Previous investigations have demonstrated the superiority of combined imaging approaches over single-modality analyses, particularly for improving T staging, lymph node assessment, and outcome prediction [36,41,42]. Reported improvements in discriminative performance, frequently reflected by higher area under the receiver operating characteristic curve (AUC) values, support the concept that integrating CT, MRI, PET, and clinical information provides a more comprehensive representation of tumor biology. Beyond improving predictive performance, multimodal approaches facilitate the characterization of complementary biological dimensions of the disease, including anatomical extent, tissue composition, vascular characteristics, and metabolic behavior. This multidimensional assessment has the potential to support more accurate patient stratification and individualized therapeutic decision-making [45]. These findings further suggest that the benefit of multiparametric radiomics arises from integrating complementary biological information across imaging modalities rather than simply increasing the number of extracted radiomic features. Accordingly, future model development should prioritize biologically meaningful, reproducible, and clinically interpretable biomarkers instead of maximizing feature dimensionality.

Despite promising results, evidence for the superiority of multiparametric radiomics remains inconsistent. While some studies report improved performance by combining CT, MRI, and PET features, others show only marginal gains over well-designed single-modality models. This inconsistency is compounded by small retrospective cohorts, heterogeneous imaging and analytical workflows, and limited external validation, raising concerns about overfitting and generalizability. Thus, increasing model complexity does not necessarily translate into greater clinical value. Future research should prioritize methodological rigor, prospective multicenter validation, and demonstration of clear incremental benefit over simpler models.

**Figure 4:**
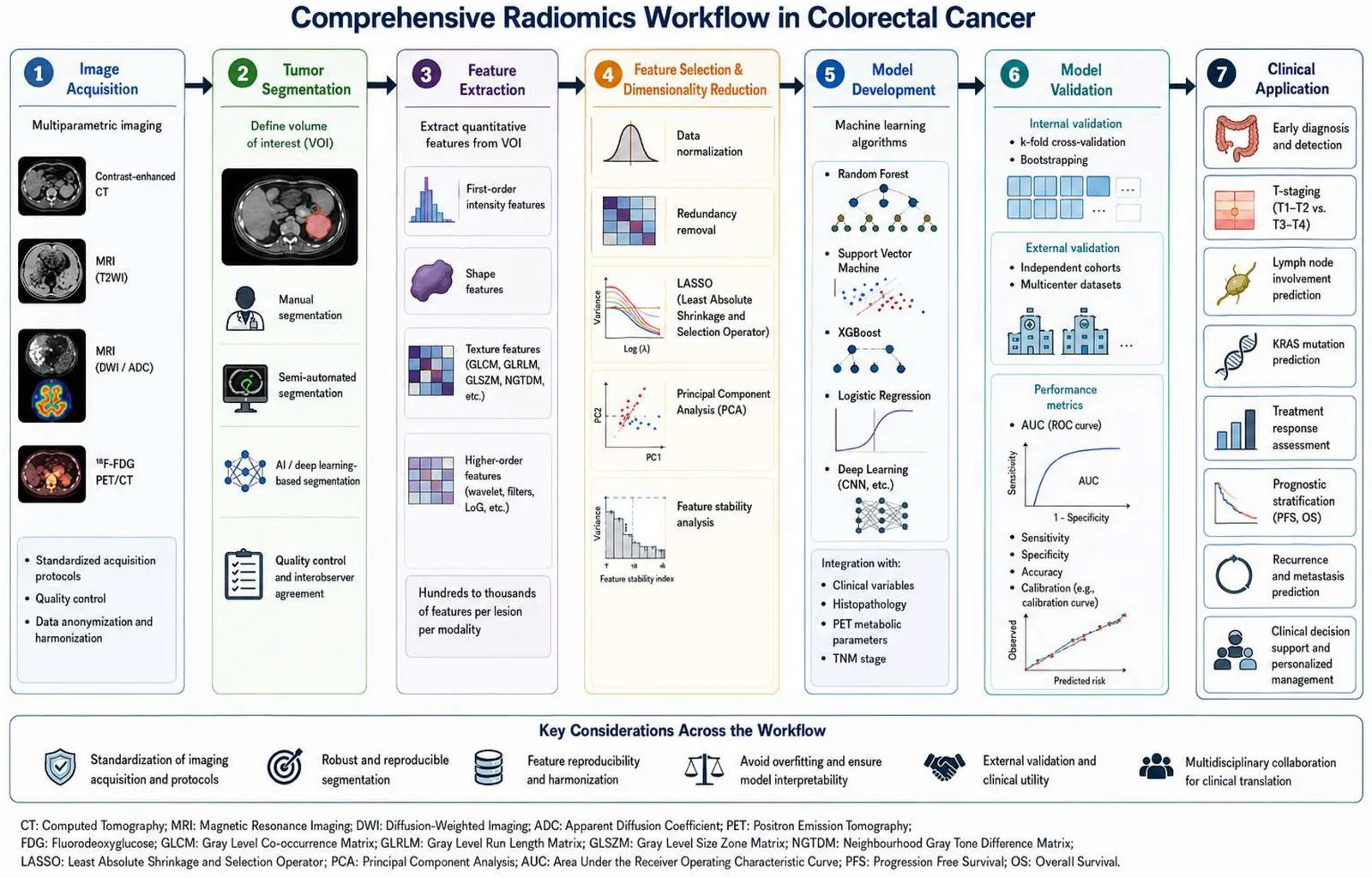
Conceptual overview of the complete multiparametric radiomics workflow in colorectal cancer, illustrating standardized multimodal image acquisition, tumor segmentation, radiomic feature extraction, feature selection and dimensionality reduction, predictive model development, validation, and translation into clinical decision-making.

This review also places particular emphasis on methodological rigor, highlighting the importance of feature selection, validation strategies, and reproducibility—areas that have been addressed inconsistently in previous studies [45,46]. The increasing complexity of radiomics models requires careful management of high-dimensional data to minimize redundancy, instability, and overfitting. Consequently, robust feature reduction techniques, transparent model development workflows, and rigorous internal and external validation are essential prerequisites for successful clinical implementation. Furthermore, model explainability and interpretability have become increasingly important as radiomics continues to converge with machine learning and artificial intelligence methodologies (Figure 4) [29,30,31,38,46].

Despite these encouraging developments, several limitations should be acknowledged. A recurring challenge across published studies is the relatively small sample size, which may reduce statistical power, increase the risk of overfitting, and consequently limit model generalizability. In addition, heterogeneity in imaging acquisition protocols, reconstruction parameters, and analytical workflows—including factors specific to PET quantification, such as standardized uptake value (SUV) variability—introduces technical variability that may affect radiomic feature stability and hinder comparisons across studies. Collectively, these limitations emphasize the need for standardized methodologies and larger, well-curated multicenter datasets [6,14,17,18,19,20,21,31].

Another important limitation is the retrospective design of many published investigations, which may introduce selection bias and reduce the clinical applicability of the reported findings. Differences in patient populations, treatment regimens, disease stage, and follow-up protocols further complicate comparisons among studies. Moreover, the robustness of radiomic features remains sensitive to segmentation variability, particularly when manual contouring is used [29]. These methodological challenges highlight the need for harmonized imaging acquisition, segmentation, and analytical frameworks capable of supporting reliable multicenter investigations and facilitating broader clinical implementation [23,26,27,34].

This review is also subject to several limitations. First, as a narrative review, it did not include a formal meta-analysis; therefore, a quantitative synthesis of diagnostic and prognostic performance metrics was not performed. Second, although a structured literature search was conducted, the study selection process may be susceptible to selection bias because article inclusion involved qualitative assessment of relevance. Third, only studies published in English were included, potentially introducing language bias and excluding relevant evidence published in other languages. Furthermore, the substantial methodological heterogeneity among the included studies with respect to imaging protocols, segmentation approaches, feature extraction methods, machine learning algorithms, and validation strategies limited direct comparisons across investigations. Finally, publication bias cannot be excluded because studies reporting positive findings are generally more likely to be published than those with negative or inconclusive results. These limitations should be considered when interpreting the conclusions of this review.

Looking forward, the integration of artificial intelligence (AI), particularly deep learning techniques, is expected to further expand the capabilities of radiomics. AI-driven approaches have the potential to automate feature extraction, optimize model development, and identify complex non-linear relationships within imaging data that are difficult to detect using conventional analytical methods [38,45,46]. Unlike handcrafted radiomic features, deep learning models can learn hierarchical image representations directly from imaging data, potentially capturing subtle imaging patterns associated with tumor biology and treatment response. As computational resources and the availability of annotated imaging datasets continue to increase, these techniques are expected to play an increasingly important role in both radiomics research and clinical implementation. Rather than replacing radiologists, AI-driven radiomics is likely to function primarily as a clinical decision-support tool by assisting image interpretation, reducing interobserver variability, and increasing diagnostic confidence in challenging clinical cases [24,27].

The combination of AI with multimodal imaging, including PET, may further enable fully automated end-to-end predictive frameworks. Such systems could integrate image acquisition, automated segmentation, feature extraction, risk prediction, and clinical decision support into a unified workflow, thereby reducing observer dependency and improving operational efficiency. Furthermore, the integration of radiogenomic and multi-omics data may considerably expand the scope of radiomics by linking imaging phenotypes with molecular, genetic, and transcriptomic characteristics, ultimately providing a more comprehensive understanding of tumor biology [35,44]. A particularly relevant example is the prediction of KRAS mutation status from routine CT, MRI, or PET imaging. Currently, KRAS testing requires dedicated molecular pathological analysis, which is associated with additional costs and tissue sampling. Several radiomics studies have demonstrated promising results in identifying imaging signatures associated with KRAS mutations, highlighting the potential of radiomics as a non-invasive surrogate biomarker [47,48].

The future clinical adoption of radiomics will depend heavily on large multicenter validation studies capable of confirming model robustness, reproducibility, and generalizability across diverse patient populations and imaging environments [22,25]. International collaborative initiatives, standardized reporting frameworks, and harmonized imaging protocols will be essential for establishing radiomics as a reliable clinical tool. If these challenges can be successfully addressed, multiparametric radiomics integrating CT, MRI, and PET has the potential to become a cornerstone of precision oncology in colorectal cancer by supporting earlier diagnosis, more accurate prognostication, individualized treatment selection, and ultimately improved patient outcomes [21,24,25,26,27,28,29,30,31,32,40,41,42,43,44,45,46].

Although radiomics has demonstrated considerable promise as a tool for precision oncology, its successful translation into routine clinical practice depends not only on predictive performance but also on methodological rigor, transparency, and reproducibility. The increasing complexity of radiomics and artificial intelligence research has highlighted the need for standardized frameworks that ensure consistent feature extraction, reliable predictive modeling, and transparent reporting. Consequently, adherence to internationally recognized guidelines, including the Image Biomarker Standardization Initiative (IBSI), Transparent Reporting of a Multivariable Prediction Model for Individual Prognosis or Diagnosis (TRIPOD), Checklist for Artificial Intelligence in Medical Imaging (CLAIM), and the Radiomics Quality Score (RQS), has become increasingly important throughout the radiomics workflow (Table 1). Collectively, these complementary frameworks address different aspects of study design, methodological quality, data analysis, reporting, and quality assessment, thereby improving the credibility, reproducibility, and clinical applicability of radiomics research [49,50,51,52].

## 9. Conclusions

Multiparametric radiomics, founded on advanced diagnostic imaging, represents a transformative approach to the management of colorectal cancer [7,8]. By integrating quantitative information derived from multimodal imaging, this approach enables a more comprehensive characterization of tumor phenotype, biological behavior, and intratumoral heterogeneity than conventional image interpretation alone [7,8,21,22].

Multiparametric radiomics has demonstrated considerable potential to improve diagnostic accuracy by identifying subtle imaging patterns beyond the limits of human visual perception, thereby enhancing tumor characterization and staging. In addition, it may facilitate personalized treatment strategies by integrating imaging biomarkers with established clinical and pathological variables to support individualized therapeutic decision-making [21,29]. Furthermore, its ability to characterize tumor heterogeneity and the tumor microenvironment suggests significant value for prognostic modeling, enabling more accurate risk stratification for progression-free and overall survival in selected patient populations [22,23,31,37].

Despite these promising findings, the current evidence should be interpreted with caution. The widespread clinical implementation of multiparametric radiomics remains constrained by the lack of standardized imaging acquisition and analysis protocols, substantial methodological heterogeneity among published studies, the predominance of retrospective single-center investigations, limited external validation, and the persistent risk of model overfitting. Together, these limitations continue to affect the reproducibility, robustness, and generalizability of published radiomics models.

Nevertheless, the available evidence indicates that multiparametric radiomics has considerable potential to become an integral component of precision oncology by bridging the gap between advanced imaging and individualized patient care. Achieving routine clinical implementation will require standardized imaging protocols, harmonized radiomics workflows, large prospective multicenter validation studies, and the development of robust, transparent, and clinically interpretable predictive models. Furthermore, radiogenomics represents a particularly promising future direction, as imaging-derived radiomic features may enable the non-invasive prediction of clinically relevant molecular alterations, including KRAS mutation status, directly from routine CT, MRI, and PET examinations. Continued progress in these areas will be essential to fully realize the clinical impact of multiparametric radiomics in colorectal cancer.

## Figures and Tables

**Table 1:** Summary of internationally recognized guidelines and quality assessment frameworks used throughout the radiomics workflow to enhance standardization, reproducibility, methodological quality, and transparent reporting of radiomics and artificial intelligence studies.

Guideline	Purpose	When to Use
IBSI (Image Biomarker Standardization Initiative)	Standardizes the calculation and definition of radiomic features to ensure reproducibility across software platforms.	During image preprocessing, segmentation, and radiomic feature extraction.
TRIPOD (Transparent Reporting of a Multivariable Prediction Model for Individual Prognosis or Diagnosis)	Provides guidance for transparent reporting of prediction model development and validation studies.	When developing, validating, or updating predictive models.
CLAIM (Checklist for Artificial Intelligence in Medical Imaging)	Provides a reporting checklist for artificial intelligence studies in medical imaging, particularly machine learning and deep learning.	When conducting and reporting AI-based medical imaging research.
RQS (Radiomics Quality Score)	Assesses the methodological quality and scientific rigor of radiomics studies.	During study design, quality assessment, and manuscript preparation.

## Data Availability

No new data were created or analyzed in this study. Data sharing is not applicable to this article.

## Supplementary Materials

The following supporting information can be downloaded at: https://www.mdpi.com/article/10.3390/cancers18162578/s1. References [13,14,16,18,19,23,24,27,28,29,30,31,32,33,34,35,36,37,38,39,41,42,43,44,45,46,47,48,49] are cited in the Supplementary Materials.

## Abbreviations

The following abbreviations are used in this manuscript:

ADC	Apparent Diffusion Coefficient
AI	Artificial Intelligence
AUC	Area Under the Curve
CLAIM	Checklist for Artificial Intelligence in Medical Imaging
CRC	Colorectal Carcinoma
CT	Computed Tomography
DWI	Diffusion-Weighted Imaging
EARL	European Association of Nuclear Medicine Research Ltd.
FDG	Fluorodeoxyglucose
GLCM	Gray-Level Co-occurrence Matrix
GLRLM	Gray-Level Run Length Matrix
IBSI	Image Biomarker Standardization Initiative
ICC	Intraclass Correlation Coefficient
LASSO	Least Absolute Shrinkage and Selection Operator
MRI	Magnetic Resonance Imaging
MTV	Metabolic Tumor Volume
OS	Overall Survival
PCA	Principal Component Analysis
PET	Positron Emission Tomography
PET/CT	Positron Emission Tomography/Computed Tomography
PET/MRI	Positron Emission Tomography/Magnetic Resonance Imaging
PFS	Progression-Free Survival
RQS	Radiomics Quality Score
SUV	Standardized Uptake Value
SUVmax	Maximum Standardized Uptake Value
SUVmean	Mean Standardized Uptake Value
SUVpeak	Peak Standardized Uptake Value
TLG	Total Lesion Glycolysis
TNM	Tumor, Node, Metastasis (staging system)
TRIPOD	Transparent Reporting of a Multivariable Prediction Model for Individual Prognosis or Diagnosis
